# Scenario-specific aberrations of social reward processing in dimensional schizotypy and psychopathy

**DOI:** 10.1038/s41598-022-18863-9

**Published:** 2022-12-13

**Authors:** Luke Aldridge-Waddon, Martina Vanova, Leonie Elbers, Ignazio Puzzo, Jaap Munneke, Veena Kumari

**Affiliations:** 1 Division of Psychology, Department of Life Sciences, College of Health, Medicine and Life Sciences, Brunel University of London, London, UK; 2 Department of Psychology, University of Wuppertal, Wuppertal, Germany; 3 Centre for Cognitive Neuroscience, College of Health, Medicine and Life Sciences, Brunel University of London, London, UK

**Keywords:** Psychology, Human behaviour

## Abstract

The feelings of reward associated with social interaction help to motivate social behaviour and influence preferences for different types of social contact. In two studies conducted in a general population sample, we investigated self-reported and experimentally-assessed social reward processing in personality spectra with prominent interpersonal features, namely schizotypy and psychopathy. Study 1 (n = 154) measured social reward processing using the Social Reward Questionnaire, and a modified version of a Monetary and Social Incentive Delay Task. Study 2 (n = 42; a subsample of Study 1) investigated social reward processing using a Social Reward Subtype Incentive Delay Task. Our results show that schizotypy (specifically Cognitive-Perceptual dimension) and psychopathy (specifically Lifestyle dimension) are associated with diverging responses to social scenarios involving large gatherings or meeting new people (Sociability), with reduced processing in schizotypy and heightened processing in psychopathy. No difference, however, occurred for other social scenarios—with similar patterns of increased antisocial (Negative Social Potency) and reduced prosocial (Admiration, Sociability) reward processing across schizotypy and psychopathy dimensions. Our findings contribute new knowledge on social reward processing within these personality spectra and, with the important exception of Sociability, highlight potentially converging patterns of social reward processing in association with schizotypy and psychopathy.

## Introduction

Social interactions have the potential to be rewarding^1^. The rewards experienced during social interaction, such as feelings of warmth and satisfaction, are associated with increased activity within neural reward networks^2^ and positive changes in subjective feelings of enjoyment and pleasure^3^. The feelings of reward extracted during social interaction appear to activate overlapping behavioural and neural mechanisms to those involved in non-social rewards (e.g., monetary rewards^3^) and, as with other reward types, social reward processing can be parsed into anticipatory and consummatory phases^4^.

Classifications of social reward^5^ outline six social reward subtypes: Admiration, Negative Social Potency, Passivity, Prosocial Interactions, Sexual Relationships and Sociability. Admiration is the receiving of praise, flattery, or positive attention from others, and its reward value has been illustrated in research using social media ‘Likes’ and positive verbal feedback^6,7^. Negative Social Potency is the enjoyment of witnessing or causing cruelty to others and has been found to be a motivating mechanism in antisocial behaviours such as internet trolling^8^. Passivity involves extracting feelings of reward from situations in which others take the lead. It correlates with self-reported submissiveness^5^ and is potentially related to social loafing^9^. The Prosocial Interactions subtype captures the warm glow hypothesis of altruism^10^ and highlights prosocial behaviours (e.g., charitable giving, emotional closeness, fairness) can be rewarding for the actor in addition to the recipient^11,12^. The Sexual Relationships subtype refers to the enjoyment of casual sexual relationships and is linked to risky sexual behaviour^13^. Finally, Sociability captures the enjoyment of social scenarios and social events, and it has been proposed^5^ that individuals who extract greater feelings of reward from Sociability are more likely to be extroverted and more motivated to engage with the wider social environment^14^.

The description of the social reward subtypes given above highlights the multiple ways in which social interaction can be rewarding. However, if reward processing mechanisms are interrupted or adjusted, it could mean that social rewards are experienced in an atypical way, leading to atypical social behaviour^15^. Our review^16^ identified that social reward processing is affected across psychiatric diagnoses, and the present study aims to follow this review and dimensionally examine two psychopathological personality continuums which may be associated with atypical social reward processing, and subsequent atypical interpersonal behaviour, namely schizotypal and psychopathic traits.

Schizotypy includes positive (unusual experiences or beliefs), negative (lower motivation, feelings of low mood), and disorganised (odd or eccentric behaviours) behaviour dimensions^17^. The negative dimension is most often associated with atypical social behaviour, such as a lack of engagement with social norms and lower motivation to be part of the social environment^18^, with the disorganised dimension linked to heightened impulsivity and novelty-seeking. Psychopathy includes a cluster of dimensional traits such as a lack of empathy, superficial charm, and sensation-seeking^19^ and, from an interpersonal perspective, is associated with antisocial attitudes, manipulativeness, and interpersonal gregariousness^20^.

Existing work testing links between these traits and social reward processing has evidenced reduced responsivity to praise in schizotypy^21^, heightened feelings of social anhedonia related to reduced wanting of social interaction across the schizotypy continuum^22^, and increased subjective experience of Admiration and Negative Social Potency in dimensional psychopathy^9^. However, no work to-date has investigated similarities in social reward processing across these dimensions. Proponents of the Eysenckian psychoticism-psychopathy continuum^23,24^ suggest that dimensions of schizotypy and psychopathy may share some behavioural features, for example increased propensity towards impulsivity^25^, with psychoticism also associated with behaviours traditionally linked to primary psychopathy (such as reduced affective empathy, reduced anxiety, and reduced punishment sensitivity^26^). It is therefore important to explore whether the shared behavioural features of the psychoticism-psychopathy continuum translate specifically to the processing of social rewards.

Within this, the subtype of social reward available is likely to be important. Some conceptualisations of psychopathy and schizotypy include a heightened proclivity for antisocial behaviour^25,27,28^, and thus we might expect both to be dimensionally associated with increased processing of ‘antisocial’ rewards i.e., Negative Social Potency. In contrast, the schizophrenia spectrum—which includes schizotypy—captures an increased tendency towards interpersonally submissive/passive behaviours^29^ and social anxiety^30^, whereas psychopathy is associated with less submissive/anxious, more bold, interpersonal behaviours^9,31^. This difference in interpersonal style may therefore reflect a social reward processing difference, although this is currently unknown based on existing research.

Thus, this paper aims to explore converging and diverging relationships between schizotypal and psychopathic traits and social reward processing. It details two investigations of social reward processing using self-report and experimental measures in a general population sample. Study 1 examined associations between these traits and scores on the Social Reward Questionnaire^5^ (SRQ) and the Monetary and Social Incentive Delay Task (MSIDT). Study 2 studied a subsample of participants from Study 1 and explored relationships between schizotypal and psychopathic traits and behavioural processing of the social reward subtypes^5^, indexed using the Social Reward Subtype Incentive Delay Task (SRS-IDT).

## Results

### Study 1

#### Self-reported social reward processing

The zero-order spearman’s rho rank correlations between schizotypal traits, psychopathic traits, and responses on the SRQ are presented in Table 1. The Cognitive-Perceptual dimension of schizotypy negatively correlated with scores on the Sociability subscale of the SRQ, *r*_s_(149) = − 0.17, *p* = 0.037, as did the Interpersonal dimension, *r*_s_(149) = − 0.39, *p* ≤ 0.001, suggesting reduced subjective processing of social rewards involving Sociability linked to these dimensions of schizotypy. In contrast to this reduced subjective processing of ‘prosocial’ rewards, associations were observed between Cognitive-Perceptual and Disorganised dimensions and increased self-reported processing of Negative Social Potency, *r*_s_(150) = 0.30, *p* ≤ 0.001, and *r*_s_(150) = 0.36, *p* ≤ 0.001, respectively, perhaps indicating that these dimensions are associated increased feelings of ‘antisocial’ reward when enacting or observing cruelty towards others. A relationship between the Disorganised dimension and increased self-reported processing of social rewards involving Sexual Relationships was also observed, *r*_s_(147) = 0.28, *p* = 0.001. No other significant associations between schizotypal traits and self-reported social reward processing were found.

**Table 1:** Correlations between SRQ scores and schizotypy and psychopathy dimensions.

	Admiration	Negative social potency	Passivity	Prosocial interactions	Sexual relationships	Sociability
**Schizotypal traits**						
Cognitive-perceptual	0.035	0.298**	0.068	− 0.050	0.068	− 0.170*
Interpersonal	− 0.140	0.147	0.065	− 0.100	− 0.079	− 0.392**
Disorganised	0.094	0.358**	0.059	− 0.116	0.276**	0.008
**Psychopathic traits**						
Interpersonal	0.026	0.601**	0.172*	− 0.253**	0.277**	0.050
Affective	− 0.175*	0.450**	0.077	− 0.291**	0.200*	− 0.095
Lifestyle	− 0.021	0.533**	− 0.022	− 0.267**	0.336**	0.173*
Antisocial	− 0.031	0.492**	0.089	− 0.221**	0.104	0.036
Total	− 0.066	0.629**	0.095	− 0.301**	0.289**	0.051

*Significant at *p* = 0.05; **Significant at *p* = 0.01; all values *r*_s_.

Several associations between psychopathic traits and self-reported social reward processing were observed. All psychopathy SRP-4-SF dimensions (and SRP-4-SF total score) correlated with increased self-reported processing of social rewards involving Negative Social Potency [Interpersonal: *r*_s_(150) = 0.60, *p* ≤ 0.001; Affective: *r*_s_(150) = 0.45, *p* ≤ 0.001; Lifestyle: *r*_s_(150) = 0.53, *p* ≤ 0.001; Antisocial: *r*_s_(150) = 0.49, *p* ≤ 0.001; total: *r*_s_(150) = 0.63, *p* ≤ 0.001]. In addition to Negative Social Potency, several dimensions were related to increased self-reported processing of Sexual Relationships [Interpersonal: *r*_s_(147) = 0.28, *p* = 0.001; Affective: *r*_s_(147) = 0.20, *p* = 0.014; Lifestyle: *r*_s_(147) = 0.34, *p* ≤ 0.001; total: *r*_s_(147) = 0.29, *p* ≤ 0.001]. A positive correlation between the Lifestyle dimension and self-reported processing of Sociability was also observed, *r*_s_(149) = 0.17, *p* = 0.034. In contrast to increased enjoyment of these subtypes of social reward in dimensional psychopathy, negative associations between all psychopathy dimensions and subjective processing of social rewards involving Prosocial Interactions were found [Interpersonal: *r*_s_(150) = − 0.25, *p* = 0.002; Affective: *r*_s_(150) = − 0.29, *p* ≤ 0.001; Lifestyle: *r*_s_(150) = − 0.27, *p* = 0.001; Antisocial: *r*_s_(150) = − 0.22, *p* = 0.006; total: *r*_s_(150) = − 0.30, *p* ≤ 0.001]. The Affective dimension was also found to negatively correlate with self-reported processing of Admiration, *r*_s_(150) = − 0.18, *p* = 0.031. Taken together, these associations highlight that social reward processing in dimensional psychopathy may be dependent on the social reward subtype available, with heightened processing of Negative Social Potency, Sociability, and Sexual Relationships, but reduced processing of Prosocial Interactions.

The regression model predicting self-reported processing of Sociability included the Cognitive-Perceptual and Interpersonal dimensions of schizotypy and the Lifestyle dimension of psychopathy. The model was statistically significant and explained 20% of the variance, *F* (3, 147) = 12.52, *p* < 0.001, *r* = 0.45, *r*^2^ = 0.20, with the Interpersonal dimension significantly predicting reduced self-reported processing of Sociability, *B* = − 0.19, *SE B* = 0.04, *β* = − 0.41, *p* < 0.001, but the Lifestyle dimension significantly predicting increased self-reported processing of Sociability, *B* = 0.19, *SE B* = 0.06, *β* = 0.25, *p* = 0.001. The model predicting self-reported processing of Negative Social Potency, which included Cognitive-Perceptual and Disorganised schizotypy dimensions and the four dimensions of psychopathy, was statistically significant and explained 39% of the variance, *F* (6, 145) = 15.70, *p* < 0.001, *r* = 0.63, *r*^2^ = 0.39. The Interpersonal and Antisocial dimensions were the only significant individual predictors in the model, both predicting increased self-reported processing of Negative Social Potency, *B* = 0.31, *SE B* = 0.10, *β* = 0.33, *p* = 0.002 and *B* = 0.53, *SE B* = 0.13, *β* = 0.34, *p* ≤ 0.001, respectively. Finally, the model predicting self-reported processing of Sexual Relationships with the Disorganised dimension of schizotypy, and the Interpersonal and Lifestyle dimensions of psychopathy, was statistically significant, *F* (3, 145) = 7.32, *p* < 0.001, *r* = 0.36, *r*^2^ = 0.13, with the Lifestyle dimension emerging as the only significant predictor, *B* = 0.24, *SE B* = 0.10, *β* = 0.25, *p* = 0.018.

#### Behavioural social reward processing

To account for gender differences in reward processing^32^ and investigate interaction effects between gender and MSIDT performance, task performance analyses were first computed with gender entered as a between-subjects variable (male n = 33, female n = 98). No significant interaction effect between gender and anticipatory RT was found *F* (1.75, 226.19) = 1.29, *p* = 0.277, η_p_^2^ = 0.01. This was also the case for response accuracy, *F* (2, 258) = 2.93, *p* = 0.055, η_p_^2^ = 0.02. Gender was thus not included as a between-subjects variable within subsequent analyses.

Analysis of main effects revealed a significant main effect of reward type on anticipatory RT, *F* (1.76, 236.09) = 8.34, *p* = 0.001, η_p_^2^ = 0.06. Post hoc pairwise comparisons with Bonferroni correction applied showed this effect to be driven by significantly faster RTs towards monetary rewards (*M* = 266.74, *SD* = 98.88) in comparison to neutral stimuli (*M* = 299.80, *SD* = 117.79). However, no significant difference in RT towards social rewards versus neutral stimuli was found. A significant main effect of reward type on response accuracy was also found, *F* (2, 268) = 27.75, *p* ≤ 0.001, η_p_^2^ = 0.17. Post-hoc tests with Bonferroni correction applied found significantly greater response accuracy towards monetary rewards (*M* = 71.42, *SD* = 22.15) than neutral stimuli (*M* = 61.23, *SD* = 25.28), and social rewards (*M* = 70.80, *SD* = 20.77) than neutral stimuli (*M* = 61.23, *SD* = 25.28). Together, the RT and response accuracy data suggest that the task functioned as expected (with greater behavioural anticipation of rewards than neutral stimuli) with participants demonstrating similar levels of behavioural anticipation for both monetary and social rewards.

As presented in Table 2, testing associations between schizotypal and psychopathic traits and MSIDT task performance revealed only one marginally significant correlation between schizotypal traits and behavioural reward processing, with the Disorganised dimension correlating with reduced response accuracy towards neutral stimuli, *r*_s_(132) = − 0.17, *p* = 0.045. No other significant associations between dimensions and MSIDT performance were found. The lack of significant task-dimension associations may be a consequence of assessing social reward processing ‘globally’ rather than differentiating between social reward subtypes. This is addressed by Study 2.

**Table 2:** Correlations between MSIDT performance and schizotypy and psychopathy dimensions.

	Monetary RT	Social RT	Neutral RT	Monetary RA	Social RA	Neutral RA
**Schizotypal traits**						
Cognitive-perceptual	0.114	0.096	0.098	0.009	− 0.071	− 0.019
Interpersonal	0.043	0.065	0.046	0.009	− 0.063	− 0.016
Disorganised	− 0.124	− 0.057	0.004	− 0.086	− 0.109	− 0.174*
**Psychopathic traits**						
Interpersonal	− 0.017	0.000	0.065	− 0.123	− 0.168	− 0.163
Affective	0.036	− 0.001	− 0.081	− 0.159	− 0.098	− 0.079
Lifestyle	− 0.058	− 0.100	− 0.022	− 0.047	0.010	− 0.101
Antisocial	0.093	0.091	0.061	− 0.100	− 0.053	− 0.031
Total	0.005	− 0.023	0.009	− 0.111	− 0.081	− 0.121

*RT* reaction time, *RA* response accuracy.

*Significant at *p* = 0.05.

### Study 2

#### Task effects

A significant main effect of social reward subtype on anticipatory RT was found, *F* (4, 164) = 7.11, *p* ≤ 0.001, η_p_^2^ = 0.15. Post hoc pairwise comparisons with Bonferroni correction applied revealed several significant differences in processing of social reward subtypes. We observed significantly faster RTs towards social rewards involving Admiration (*M* = 247.23, *SD* = 121.08) in comparison to Negative Social Potency (*M* = 359.87, *SD* = 168.56) and Passivity (*M* = 315.86, *SD* = 162.78). Similarly, RTs towards social rewards involving Sociability (*M* = 267.40, *SD* = 125.64) were significantly faster than those towards Negative Social Potency. This indicates that Admiration and Sociability may have been the most salient social reward subtypes and/or that Negative Social Potency may have been less salient than other social reward subtypes. A significant main effect of social reward type was also found for anticipatory response accuracy, *F* (2.41, 98.70) = 8.07, *p* ≤ 0.001, η_p_^2^ = 0.16. As with RT, post-hoc analysis found that anticipatory response accuracy towards Negative Social Potency (*M* = 54.56, *SD* = 28.47) was significantly lower than all social reward subtypes other than Sociability. Taken together, these results indicate that social rewards involving Negative Social Potency may be less incentivising than other social reward subtypes for most individuals, which is perhaps to be expected in a general population sample given that Negative Social Potency is defined as the enjoyment of witnessing or enacting cruelty to others.

#### Associations with schizotypal and psychopathic traits

As presented in Table 3, the Cognitive-Perceptual dimension of the schizophrenia spectrum significantly correlated with reduced behavioural processing of social rewards involving Admiration, as indexed by slower anticipatory RTs, *r*_s_(40) = 0.36, *p* = 0.020, and reduced processing of Sociability, reflected in slower anticipatory RTs, *r*_s_(40) = 0.38, *p* = 0.013. In addition to reduced processing of these social reward types, the Cognitive-Perceptual dimension correlated with increased response accuracy towards social rewards involving Passivity, *r*_s_(40) = 0.32, *p* = 0.042, and the Disorganised dimension significantly correlated with reduced processing (slower anticipatory RTs) towards social rewards involving Negative Social Potency, *r*_s_(40) = 0.34, *p* = 0.029.

**Table 3:** Correlations between SRS-IDT performance and schizotypy and psychopathy dimensions.

	Admiration RT	Negative social potency RT	Passivity RT	Sociability RT	Neutral RT	Admiration RA	Negative social potency RA	Passivity RA	Sociability RA	Neutral RA
**Schizophrenia spectrum traits**										
Cognitive-perceptual	0.357*	0.229	− 0.020	0.382*	0.303	− 0.121	0.256	0.316*	− 0.003	− 0.140
Interpersonal	0.041	0.145	− 0.085	0.047	0.191	0.040	0.135	0.284	0.167	0.058
Disorganised	0.260	0.336*	0.034	0.285	0.292	0.148	0.069	0.200	0.282	0.129
**Psychopathic traits**										
Interpersonal	− 0.176	0.033	− 0.128	− 0.010	− 0.085	− 0.124	− 0.068	0.046	− 0.141	− 0.056
Affective	− 0.239	− 0.006	− 0.264	− 0.123	− 0.181	− 0.087	0.074	0.058	− 0.077	0.030
Lifestyle	− 0.288	− 0.220	− 0.287	− 0.337*	− 0.291	− 0.226	− 0.056	− 0.072	− 0.140	− 0.084
Antisocial	0.070	− 0.076	− 0.087	0.253	0.095	− 0.311*	0.181	0.060	− 0.270	− 0.208
Total	− 0.219	− 0.068	− 0.255	− 0.131	− 0.164	− 0.221	− 0.011	0.001	− 0.185	− 0.079

*RT* reaction time; *RA* response accuracy.

*Significant at p = 0.05.

As shown in Table 3, a negative association between anticipatory response accuracy towards Admiration and the Antisocial dimension of psychopathy was observed, *r*_s_(40) = − 0.31, *p* = 0.045; indicating that this dimension of psychopathy might be associated with reduced processing of social rewards involving Admiration. In contrast, the Lifestyle dimension was associated with increased processing of Sociability, correlating with faster RTs towards rewards involving Sociability, *r*_s_(40) = − 0.34, *p* = 0.029.

The only SRS-IDT metric that was significantly related to both schizotypy and psychopathy dimensions was RT towards social rewards involving Sociability. The model included the Cognitive-Perceptual dimension of schizotypy and the Lifestyle dimension of psychopathy, but was not statistically significant, *F* (2, 39) = 2.88, *p* = 0.068, *r* = 0.36, *r*^2^ = 0.13.

As a supplementary analysis, we examined zero-order spearman’s rank correlations between SRS-IDT metrics and corresponding SRQ subscales (Admiration, Negative Social Potency, Passivity, Sociability). We observed a statistically significant correlation between faster RTs towards Sociability (SRS-IDT) and Sociability subscale scores, *r*_s_(40) = − 0.33, *p* = 0.035. No other significant correlations between SRS-IDT metrics and corresponding subscales were observed—although RTs towards Neutral stimuli were found to significantly correlate with both Admiration, *r*_s_(40) = − 0.33, *p* = 0.033, and Sociability, *r*_s_(40) = − 0.36, *p* = 0.021, scores. A full table of correlation values is provided in the supplementary information (Table S5).

## Discussion

The two studies presented here explored associations between schizotypal traits, psychopathic traits, and social reward processing. Social reward processing has been assessed using the SRQ and two experimental tasks, namely the MSIDT and SRS-IDT. Study 2 tested a subset of participants who took part in Study 1.

Dealing first with task main effects, the analysis of MSIDT performance (Study 1) revealed a main effect of reward type on RT and response accuracy, with significantly greater anticipatory response accuracy towards social and monetary rewards then neutral stimuli, as well as significantly faster RTs towards monetary rewards than neutral stimuli. Similarly, the analysis of SRS-IDT performance (Study 2) revealed a main effect of reward type, perhaps suggesting that Negative Social Potency may be less incentivising than other social reward subtypes in a general population sample. Together, these findings suggest the tasks had the intended effect: they elicited reward responses at the behavioural level, for example eliciting greater behavioural processing during reward rather than non-reward trials (MSIDT, Study 1). As such, the tasks employed here illustrate the potential for integrating dynamic social reward stimuli (such as avatar animations) within reward paradigms and thus may have value in future social reward studies.

Across studies, most relationships between schizotypy and social reward processing focused on the Cognitive-Perceptual dimension. Given that atypical social behaviour in schizotypy is conceptually most linked to the Interpersonal dimension^33^, it is perhaps surprising that the Cognitive-Perceptual dimension, rather than the Interpersonal dimension, was associated with reduced social reward processing—particularly social rewards involving Sociability (Study 1 and 2) and Admiration (Study 2). One potential explanation is that this relationship is driven by the subordinate features of Cognitive-Perceptual symptomatology, namely ideas of reference and a tendency towards suspiciousness. Ideas of reference share some phenotypic similarities with social anxiety^34^, with individuals who self-report more ideas of reference expressing increased anxiety about other people judging them or being laughed at by others^35^. Therefore, it is perhaps logical that more prominent ideas of reference would lead to reduced processing of social rewards involving Sociability or Admiration; subtypes of social reward which inherently involve being paid attention to or increase ones’ exposure to the judgements of others^5^.

This pattern of reduced processing of social rewards involving Sociability linked to the Cognitive-Perceptual dimension of schizotypy is contrasted by the positive associations observed between Sociability and the Lifestyle dimension of psychopathy across both studies. The Lifestyle dimension of psychopathy captures a proneness to boredom, thrill-seeking, and a tendency to behave impulsively or recklessly^31^. In an interpersonal context, an individual with marked psychopathic traits might thus appear to be lively, sociable, and, following the results presented here, extract feelings of reward from large social gatherings or meeting new people. However, the observed relationship between the Lifestyle dimension and increased processing of social rewards was not observed in other work which has examined social reward processing in psychopathy previously^9^, and thus should be replicated before attaching too much weight to its interpretation.

The remaining associations were similar across schizotypy and psychopathy dimensions. We observed increased self-reported processing of Negative Social Potency linked to Cognitive-Perceptual and Disorganised dimensions of schizotypy and all psychopathy dimensions (Study 1), as well as correlations between self-reported enjoyment of Sexual Relationships and the Disorganised dimension of schizotypy and the Interpersonal and Lifestyle dimensions of psychopathy (Study 1). Associations between Cognitive-Perceptual and Antisocial dimensions and reduced behavioural processing of Admiration were also observed (Study 2). Taken together, these findings suggest similar social reward profiles in dimensional schizotypy and psychopathy: Increased enjoyment of antisocial interactions (indexed through Negative Social Potency), increased self-reported sexual sensation seeking (indexed through Sexual Relationships), and reduced behavioural anticipation of Admiration. Following work on the psychoticism-psychopathy continuum^23^ it would be interesting for future work to see how these similar reward processing profiles are linked to overlaps in specific aspects of symptomatology. Indeed, the Disorganised and Lifestyle dimensions may jointly capture a shared proclivity for impulsive and sensation-seeking behaviour which translates to a shared enjoyment of Negative Social Potency and Sexual Relationships, for example.

This study included self-report and experimental measures of social reward processing, namely the SRQ, the MSIDT, and SRS-IDT. Combining measures in this way allowed us to thoroughly explore social reward processing across dimensional schizotypy and psychopathy. Within this, through using the SRQ, Study 1 importantly highlights that the hedonic value of social rewards in psychopathology may depend on the social reward subtype available, and this was developed in Study 2 using the SRS-IDT. To-date, this is the first incentive delay paradigm to simultaneously assess behavioural processing of different subtypes of social reward and thus its inclusion is a strength of this research. Indeed, whilst other research has examined the reward value of social reward subtypes in separate tasks, combining them within an incentive delay format has helped to study the behavioural processing of different social reward subtypes without increasing the cognitive demands of the task, as is often the case with other social interaction paradigms^2,36^.

The inclusion of avatar-based social rewards is a strength of this research. The avatar videos appear to have benefited the ecological and engagement value of the tasks^37^ and are line with reward research recommendations^38^. However, it is important to note that the avatar stimuli did not capture all social reward subtypes—Prosocial Relationships and Sexual Relationships were not included in the tasks due to difficulty developing stimuli which accurately (and ethically) reflected the social reward subtype definitions. Given that Study 1 found associations between dimensions and self-reported processing of these subtypes, future research should thus develop the tasks to include avatar-based representations of these subtypes, thereby increasing their usefulness. Similarly, whilst the use of these experimental tasks is promising, the lack of meaningful correlations between SRS-IDT metrics and SRQ scores (Table S5) reminds us that there is some way to go in the stimuli development process before finding stimuli that comprehensively reflect the social reward subtypes. It is also possible that examining both the questionnaire and experimental measures against real-life social outcomes would tell us more about how well they map-onto social reward processes. Moreover, whilst the use of avatar videos is a strength of this research, no real monetary or social rewards were attached to task performance, which could have potentially restricted the actual reward value of the MSIDT and SRS-IDT. Finally, it is important to note that many of the correlations reported are uncorrected and are small-medium in size and, following the large number of correlations computed, should thus be replicated in larger samples before being accepted conclusively.

In summary, this paper has detailed two studies investigating social reward processing in dimensional schizotypy and psychopathy. Using self-report and experimental measures of social reward processing, it has identified that schizotypy (specifically Cognitive-Perceptual dimension) and psychopathy (specifically Lifestyle dimension) may be associated with diverging reward responses to social scenarios involving large gatherings or meeting new people (Sociability), with reduced reward processing in schizotypy and heightened processing in psychopathy. The remaining social reward processing responses were similar across schizotypy and psychopathy dimensions—with similar patterns of increased antisocial (Negative Social Potency) and reduced prosocial (Admiration, Sociability) reward processing across schizotypy and psychopathy dimensions. The findings presented therefore contribute new knowledge on social reward processing within these personality spectra and, with the important exception of Sociability, highlight potentially converging patterns of social reward processing in dimensional schizotypy and psychopathy.

## Methods

### Study 1

#### Ethics

This research was approved by the College of Health, Medicine and Life Sciences Research Ethics Committee (DLS), Brunel University London (ID: 16789). The research was performed in accordance with relevant guidelines/regulations and the Declaration of Helsinki. All participants provided informed consent prior to participating in the study. All participants were compensated via university course credits (4) or an amazon voucher (£10).

#### Participants and procedure

One hundred and fifty-four participants (36 male-identifying, 114 female-identifying, 4 gender non-binary) were recruited into this study via volunteer sampling. The mean age of the sample was 21.76 (*SD* = 6.08, range = 18–55). Sixty-one percent of the sample reported English as their first language, and 71.9% of the sample reported studying at undergraduate or postgraduate levels of education. Mean sample scores on schizotypy and psychopathy measures are presented in Table S1. Exclusion criteria included: (1) evidence of current or previous mental illness diagnoses, (2) evidence of current or previous serious head injury or neurological injury, (3) current and/or recent illicit substance dependence, and (4) current use of psychotropic medications that may affect neurocognitive functioning. All exclusion criteria were assessed via self-report during participant screening. All participants completed all measures in one research session, which took place at a university psychology laboratory or online (n = 81 participated in laboratory, n = 73 participated online). After familiarising themselves with the aims and purpose of the research, participants provided informed consent and then completed the MSIDT followed by all self-report measures.

#### Measuring schizotypal and psychopathic traits

Schizotypal traits were assessed using the brief revised version of the Schizotypal Personality Questionnaire^17^ (SPQ-BR). It has 32 items which are rated on a five-point Likert scale (0 = Strongly disagree, 4 = Strongly agree), with three subscales capturing Cognitive-Perceptual, Interpersonal, and Disorganised dimensions. Psychopathic traits were assessed using the Self-Report Psychopathy Scale 4 Short Form^39^ (SRP-4-SF). It includes 29 items and collects participant responses on a five-point Likert scale (1 = Strongly disagree, 5 = Strongly agree). The scale generates a total score for overall level of psychopathic traits and includes scores for each of the four dimensions of psychopathy: Interpersonal, Affective, Lifestyle and Antisocial. Both measures are suitable for use in the general population and are structured so that higher scores indicate more pronounced traits.

The zero-order spearman’s rank correlations between SPQ-BR and SRP-4-SF scores for Study 1 are presented in the supplementary information (Table S2). Correlations (*r*_s_) ranged in size from 0.04 (SPQ-BR Interpersonal and SRP-4-SF Antisocial) to 0.44 (SPQ-BR Disorganised and SRP-4-SF Interpersonal; SPQ-BR Disorganised and SRP-4-SF Lifestyle; SPQ-BR Disorganised and SRP-4-SF Total), with all but two correlations (SPQ-BR Interpersonal and SRP-4-SF Lifestyle; SPQ-BR Interpersonal and SRP-4-SF Antisocial) reaching statistical significance.

#### Social reward questionnaire

Self-reported social reward processing was assessed via the SRQ^5^. The measure has 23 items which span the six different subtypes of social reward: Admiration, Negative Social Potency, Passivity, Prosocial Interactions, Sexual Relationships, and Sociability. Responses are collected via a seven-point Likert scale (Strongly disagree = 1, Strongly agree = 7) with higher scores suggesting heightened reward processing.

#### Monetary and social incentive delay task

**Figure 1:**
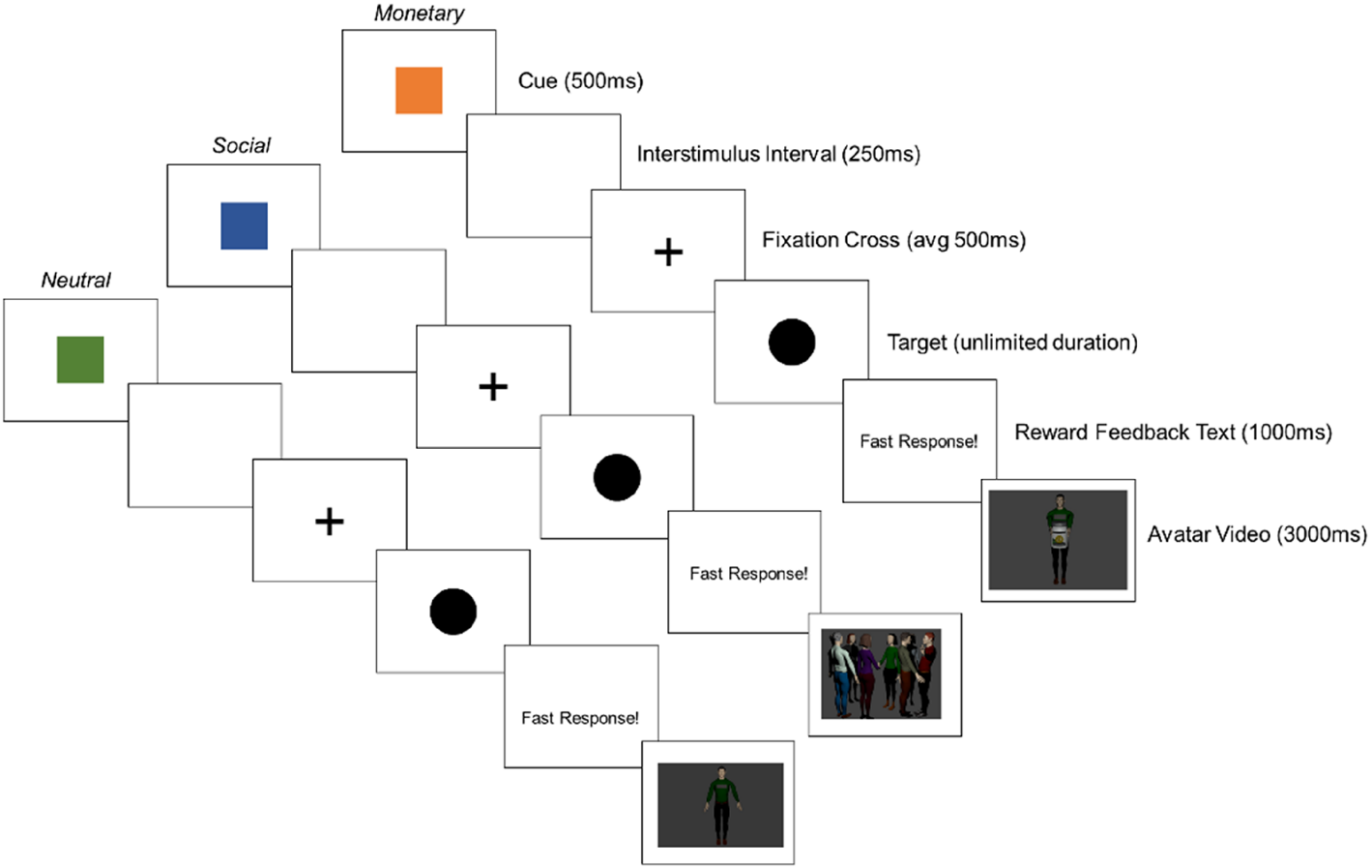
Monetary and social incentive delay task.

The MSIDT was used as a behavioural measure of social reward processing. It is designed to assess behavioural anticipation of monetary rewards, social rewards, and neutral stimuli (12 trials per stimuli type). This research employed a modified version of the traditional incentive delay paradigm in which participants could win monetary or social rewards for an avatar which were presented via animated video (Fig. 1). Participants selected which avatar they wanted to represent themselves as prior to the start of the first trial, and they were encouraged to relate to the avatar as much as possible.

Each trial began with the presentation of a cue indicating the type of reward that was available. This was followed by a target, which the participant had to respond to within the predefined RT threshold to obtain the reward. Task performance was calibrated with the participants’ individual reaction time by setting the RT threshold during the practice trials (threshold defined as mean RT across practice trials). This follows other studies^3^ that have set a bespoke RT threshold rather than setting it trial-by-trial or at a precise value (e.g., 500 ms^40^). The participant obtained the reward if they responded to the target faster than their individual RT threshold.

As illustrated in Fig. 1, each trial had a six-part sequence with a 500 ms interval between trials: (1) cue (500 ms), (2) interstimulus interval (250 ms), (3) fixation cross (jittered duration, average 500 ms), (4) target (unlimited duration), (5) reward feedback text (1000 ms), and (6) reward feedback video (3000 ms). The reward types were intermixed and there were twelve trials per reward type.

Monetary rewards showed the avatar receiving a coin into a money jar which was accompanied by the sound of a cash register. Social rewards showed the avatar engaging in four different subtypes of social reward (Admiration, Negative Social Potency, Passivity and Sociability), each accompanied by a matching sound (e.g., Admiration = Video of avatar receiving applause from a crowd, accompanied by sound of clapping and cheering). Neutral stimuli showed the avatar stood stationary in the centre of the screen and were accompanied by a neutral tone. Pixelated videos accompanied by the sound of radio static were shown following any misses, and the participant was prompted that they responded too slowly. All rewards were administered via video and no actual reward was attached to task performance. Processing of the reward types in the MSIDT was indexed through RT and response accuracy (%), with faster RTs and greater response accuracy indicating increased reward processing^41^.

#### Data analysis

All participants completed the SPQ-BR and SRP-4-SF (n = 154). However, one participant’s SPQ-BR data had to be removed due to missing values, reducing the sample size for this specific measure to n = 153. Similarly, SRQ subscale scores could not be calculated for the full sample due to missing values (Admiration: n = 152, Negative Social Potency: n = 152; Passivity: n = 152; Prosocial Interactions: n = 152; Sexual Relationships: n = 149; Sociability: n = 151). Fourteen participants provided incomplete MSIDT data and thus were not included in the task data analyses. Furthermore, participants who recorded a mean RT ≥ 1000 ms for any of the reward types were identified as outliers and thus excluded from further analyses. This affected five participants, leaving a final sample of n = 135 for task data analyses. Main effects of reward type on MSIDT task performance (n = 135) were tested via a series of ANOVAs, using RT and response accuracy per reward type (monetary, social, neutral) as the dependent variable. Gender was first entered as a between-subjects variable (male-identifying or female-identifying) in this comparison and then removed if no significant main or interaction effects were found. The Greenhouse–Geisser correction was applied when sphericity was violated as per Mauchly’s Test of Sphericity (p < 0.05). Associations between SPQ-BR and SRP-4-SF scores and reward processing (SRQ, MSIDT) were assessed via non-parametric zero-order correlations due to non-normal distribution of variables. Where significant relationships were observed across both schizotypy and psychopathy dimensions, variables significantly correlating with SRQ scores or MSIDT performance were entered into regression models to examine the shared contributions of schizotypal and psychopathic traits to social reward processing.

### Study 2

#### Ethics

This research was approved by the College of Health, Medicine and Life Sciences Research Ethics Committee (DLS), Brunel University London (ID: 25253). The research was performed in accordance with relevant guidelines/regulations and the Declaration of Helsinki. All participants provided informed consent prior to participating in the study. All participants were compensated for their time via university course credits (4).

#### Participants and procedure

This study tested a subset of participants from Study 1 (n = 42). The mean age of the sample was 19.79 (*SD* = 2.48, range = 18–34). Most of the sample (81%) identified as female and were all undergraduate students at the point of participation. Mean sample scores on schizotypy and psychopathy measures are presented in Table S3. The inclusion/exclusion criteria and procedure were applied as per Study 1.

#### Measuring schizotypal and psychopathic traits

SPQ-BR and SRP-4-SF were used to measure traits as in Study 1.

The zero-order spearman’s rank correlations between SPQ-BR and SRP-4-SF scores for Study 2 are presented in the supplementary information (Table S4). Correlations (*r*_s_) ranged in size from 0.02 (SPQ-BR Interpersonal and SRP-4-SF Antisocial) to 0.39 (SPQ-BR Cognitive-Perceptual and SRP-4-SF Interpersonal). Only three correlations (SPQ-BR Cognitive-Perceptual and SRP-4-SF Interpersonal; SPQ-BR Cognitive-Perceptual and SRP-4-SF Antisocial; SPQ-BR Disorganised and SRP-4-SF Affective) reached statistical significance.

#### Social reward subtype incentive delay task

The SRS-IDT was used to assess behavioural processing of four subtypes of social reward: Admiration, Negative Social Potency, Passivity and Sociability. The SRS-IDT assesses behavioural anticipation of these four subtypes, with 12 trials per reward type, and 12 trials for neutral stimuli. As with the MSIDT used in Study 1, all rewards were presented via video and depicted avatars engaging in the four subtypes of social reward. Participants selected which avatar they wanted to represent themselves as prior to the start of the first trial and were encouraged to relate to the avatar’s experience as much as possible.

Social rewards involving Admiration depicted the avatar as the centre of attention whilst receiving applause and recognition from others. This was accompanied by the sound of cheering and clapping. Social rewards involving Negative Social Potency showed the avatar bullying others. This included them jeering and laughing whilst pointing at a crying avatar. Social rewards involving Passivity showed the avatar on the peripheries of social interaction, watching others take control and the initiative. This was accompanied by the sound of whispering, to give the impression the avatar was not involved in the group interaction. Social rewards involving Sociability showed the avatar engaging in large group social interactions, accompanied by the sound of chatter and social activity. Neutral stimuli showed the avatar standing stationary in the centre of the screen and were accompanied by a neutral tone.

**Figure 2:**
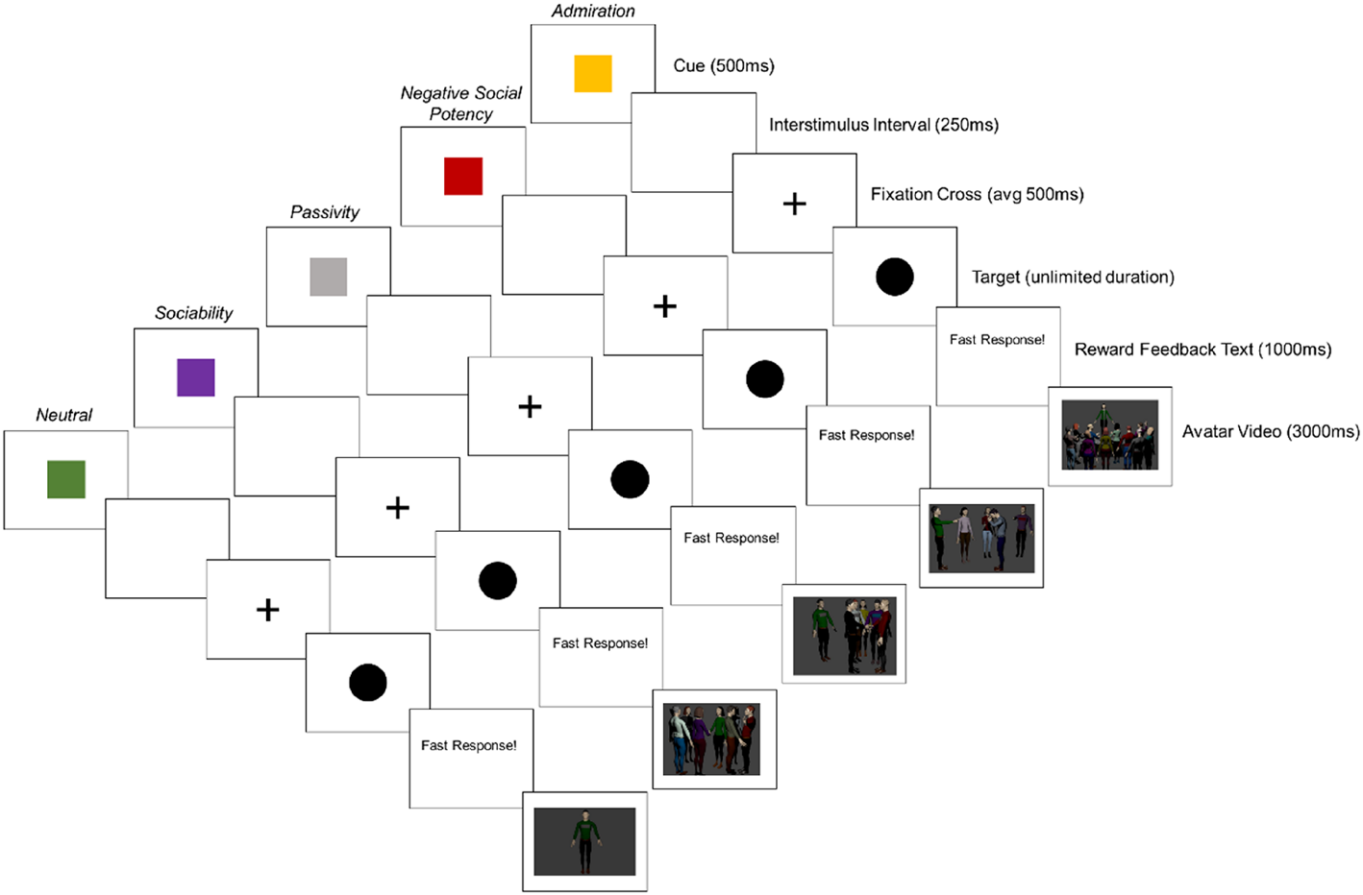
Social reward subtype incentive delay task.

Each trial began with a cue indicating which subtype of social reward was available to win. To ease accessibility and decrease working memory demands, the social reward subtype linked to the cue (e.g., Admiration) was also written on the cue. As shown in Fig. 2, each trial had a six-part sequence with a 500 ms interval between trials: (1) cue (500 ms), (2) interstimulus interval (250 ms), (3) fixation cross (jittered duration, average 500 ms), (4) target (unlimited duration), (5) reward feedback text (1000 ms), and (6) reward feedback video (3000 ms). As per the MSIDT in Study 1, winning rewards was dependent on the participant responding to their bespoke RT threshold which was set during the practice trials. Pixelated videos accompanied by the sound of radio static were shown following any misses, and the participant was prompted to respond faster to obtain the available rewards. Like the MSIDT in Study 1, processing was indexed through RT (ms) and response accuracy (%), with faster RTs and greater response accuracy indicating increased reward processing.

#### Data analysis

Main effects of social reward subtype on task performance were assessed using repeated measures ANOVAs, with RT and response accuracy per reward subtype (Admiration, Negative Social Potency, Passivity, Sociability, Neutral) as the dependent variable. Again, the Greenhouse–Geisser correction was applied when sphericity was violated as per Mauchly’s Test of Sphericity (p < 0.05). Associations between schizotypal traits, psychopathic traits, and SRS-IDT performance were explored via zero-order Spearman’s rank order correlations, again due to non-normal data distributions. As in Study 1, significant relationships observed across dimensions were entered together into regression models to examine their converging/diverging contributions to social reward processing.

## Supplementary Information

Supplementary Tables.

## Data Availability

The datasets generated and/or analysed for the investigations reported in this paper are available in the OSF repository, https://osf.io/fp6qb/, 10.17605/OSF.IO/FP6QB.
