# Supplementary material for: Scenario-specific aberrations of social reward processing in dimensional schizotypy and psychopathy
Source: Sci Rep. 2022 Dec 13;12:21536. doi: 10.1038/s41598-022-18863-9 (PMC9747960; doi:10.1038/s41598-022-18863-9)
Supplement: Supplementary file 1 — Supplementary Tables. [file 41598_2022_18863_MOESM1_ESM.docx]

**Supplementary Information**

**Table S1.**

*Study 1 Scores on Schizotypy and Psychopathy Measures.*

|  | SPQ-BR Cognitive-Perceptual | SPQ-BR Interpersonal | SPQ-BR Disorganised | SRP-4-SF Interpersonal | SRP-4-SF Affective | SRP-4-SF Lifestyle | SRP-4-SF Antisocial | SRP-4-SF Total |
| --- | --- | --- | --- | --- | --- | --- | --- | --- |
| Mean | 19.14 | 19.47 | 16.08 | 13.38 | 13.60 | 15.32 | 10.21 | 53.08 |
| SD | 10.324 | 8.478 | 7.017 | 5.343 | 4.607 | 5.190 | 3.176 | 15.470 |
| Min-Max | 0-48 | 0-39 | 0-32 | 7-29 | 7-30 | 7-29 | 7-22 | 30-97 |

**Table S2.**

*Study 1 Correlations Between Schizotypy and Psychopathy Scores.*

|  | SRP-4-SF Interpersonal | SRP-4-SF Affective | SRP-4-SF Lifestyle | SRP-4-SF Antisocial | SRP-4-SF Total |
| --- | --- | --- | --- | --- | --- |
| SPQ-BR Cognitive-Perceptual | .42 (*p* < .001) | .37 (*p* < .001) | .26 (*p* = .001) | .26 (*p* = .001) | .39 (*p <* .001) |
| SPQ-BR Interpersonal | .20 (*p* = .013) | .26 (*p* = .001) | .10 (*p* = .229) | .04 (*p* = .603) | .19 (*p* = .018) |
| SPQ-BR Disorganised | .44 (*p <* .001) | .33 (*p <* .001) | .44 (*p <* .001) | .17 (*p* = .035) | .44 (*p <* .001) |

*All values* *r*_s_

**Table S3.**

*Study 2 Scores on Schizotypy and Psychopathy Measures.*

|  | SPQ-BR Cognitive-Perceptual | SPQ-BR Interpersonal | SPQ-BR Disorganised | SRP-4-SF Interpersonal | SRP-4-SF Affective | SRP-4-SF Lifestyle | SRP-4-SF Antisocial | SRP-4-SF Total |
| --- | --- | --- | --- | --- | --- | --- | --- | --- |
| Mean | 18.55 | 20.10 | 15.10 | 11.83 | 12.93 | 13.74 | 9.69 | 49.26 |
| SD | 10.753 | 9.159 | 6.596 | 5.079 | 3.960 | 4.924 | 3.302 | 15.143 |
| Min-Max | 0-48 | 0-39 | 0-32 | 7-25 | 7-23 | 7-28 | 7-22 | 31-94 |

**Table S4.**

*Study 2 Correlations Between Schizotypy and Psychopathy Scores.*

|  | SRP-4-SF Interpersonal | SRP-4-SF Affective | SRP-4-SF Lifestyle | SRP-4-SF Antisocial | SRP-4-SF Total |
| --- | --- | --- | --- | --- | --- |
| SPQ-BR Cognitive-Perceptual | .39 (*p =* .010) | .15 (*p =* .327) | .15 (*p* = .339) | .32 (*p* = .039) | .27 (*p =* .088) |
| SPQ-BR Interpersonal | .23 (*p* = .144) | .17 (*p* = .280) | -.09 (*p* = .553) | .02 (*p* = .903) | .09 (*p* = .589) |
| SPQ-BR Disorganised | .29 (*p* = .066) | .33 (*p* = .032) | .10 (*p =* .515) | .21 (*p* = .176) | .27 (*p* = .086) |

*All values* *r*_s_

**Table S5.**

*Study 2 Correlations Between SRS-IDT metrics and Social Reward Questionnaire scores*

|  | Admiration | Negative Social Potency | Passivity | Sociability |
| --- | --- | --- | --- | --- |
| Admiration RT | -.13 (*p =* .398) | .06 (*p =* .722) | .08 (*p* = .614) | -.19 (*p* = .230) |
| Negative Social Potency RT | -.06 (*p* = .694) | -.08 (*p* = .597) | .072 (*p* = .650) | -.14 (*p* = .368) |
| Passivity RT | -.09 (*p* = .572) | -.12 (*p* = .442) | -.25 (*p =* .118) | -.23 (*p* = .152) |
| Sociability RT | -.23 (*p* = .148) | .13 (*p* = .411) | -.02 (*p =* .901) | -.33 (*p* = .035) |
| Neutral RT | -.33 (*p* = .033) | .09 (*p* = .583) | -.05 (*p =* .771) | -.36 (*p* = .021) |
|  |  |  |  |  |
| Admiration RA | -.01 (*p* = .971) | -.15 (*p* = .356) | -.05 (*p =* .778) | -.20 (*p* = .208) |
| Negative Social Potency RA | -.11 (*p* = .489) | .22 (*p* = .159) | .18 (*p =* .248) | -.21 (*p* = .185) |
| Passivity RA | -.18 (*p* = .251) | .23 (*p* = .151) | .23 (*p =* .137) | -.25 (*p* = .105) |
| Sociability RA | .04 (*p* = .828) | -.08 (*p* = .626) | -.01 (*p =* .967) | -.06 (*p* = .690) |
| Neutral RA | .02 (*p* = .916) | -.04 (*p* = .809) | -.02 (*p =* .888) | -.13 (*p* = .428) |

*All values* *r*_s_
